# LAP2 Is Widely Overexpressed in Diverse Digestive Tract Cancers and Regulates Motility of Cancer Cells

**DOI:** 10.1371/journal.pone.0039482

**Published:** 2012-06-20

**Authors:** Hyun-Jung Kim, Sun-Hwi Hwang, Myoung-Eun Han, Sungmin Baek, Hey-Eun Sim, Sik Yoon, Sun-Yong Baek, Bong-Seon Kim, Jeong-Hwan Kim, Seon-Young Kim, Sae-Ock Oh

**Affiliations:** 1 Department of Anatomy, School of Medicine, Pusan National University, Pusan, Republic of Korea; 2 Medical Research Center for Ischemic Tissue Regeneration, Pusan National University, Pusan, Republic of Korea; 3 Department of Surgery, School of Medicine, Pusan National University, Pusan, Republic of Korea; 4 Medical Genomics Research Center, KRIBB, Daejeon, Republic of Korea; University of Hong Kong, Hong Kong

## Abstract

**Background:**

Lamina-associated polypeptides 2 (LAP2) is a nuclear protein that connects the nuclear lamina with chromatin. Although its critical roles in genetic disorders and hematopoietic malignancies have been described, its expression and roles in digestive tract cancers have been poorly characterized.

**Methods:**

To examine the expression of LAP2 in patient tissues, we performed immunohistochemistry and real-time PCR. To examine motility of cancer cells, we employed Boyden chamber, wound healing and Matrigel invasion assays. To reveal its roles in metastasis in vivo, we used a liver metastasis xenograft model. To investigate the underlying mechanism, a cDNA microarray was conducted.

**Results:**

Immunohistochemistry in patient tissues showed widespread expression of LAP2 in diverse digestive tract cancers including stomach, pancreas, liver, and bile duct cancers. Real-time PCR confirmed that LAP2β is over-expressed in gastric cancer tissues. Knockdown of LAP2β did not affect proliferation of most digestive tract cancer cells except pancreatic cancer cells. However, knockdown of LAP2β decreased motility of all tested cancer cells. Moreover, overexpression of LAP2β increased motility of gastric and pancreatic cancer cells. In the liver metastasis xenograft model, LAP2β increased metastatic efficacy of gastric cancer cells and mortality in tested mice. cDNA microarrays showed the possibility that myristoylated alanine-rich C kinase substrate (MARCKS) and interleukin6 (IL6) may mediate LAP2β-regulated motility of cancer cells.

**Conclusions:**

From the above results, we conclude that LAP2 is widely overexpressed in diverse digestive tract cancers and LAP2β regulates motility of cancer cells and suggest that LAP2β may have utility for diagnostics and therapeutics in digestive tract cancers.

## Introduction

Metastasis of cancer cells greatly affects prognosis of cancer patients. Survival rate of patients who have distant metastasis is significantly lower than those who have localized tumor in most types of cancer [1]. One of critical factors in metastasis is motility of cancer cells [2]. Many critical molecules which regulate motility of cancer cells have been identified. Because inhibition of migration is effective in treating metastasis in many aspects, many migration inhibitors are under the clinical development [3]. For example, Rho kinase is a small GTPase which regulates actin and microtubulin network and cellular protrusions. So, an inhibitor which targets Rho kinase is under the clinical development [3].

Lamina-associated polypeptides 2 (LAP2) is one of LEM-domain proteins which are inner nuclear membrane proteins which share a common motif of approximately 40 amino acids, known as the LEM-domain [4], [5]. LEM-domain proteins connect the inner nuclear membrane and the nuclear lamina with chromatin through the barrier-to-autointegration factor (BAF). The family of LEM-domain proteins includes LAP2 [6], [7], [8], emerin [9], MAN1 [4], LEM2 [10] and LEM3 [11]. The name LEM derives from LAP2, Emerin and MAN1 [4].

In addition to their structural roles in nuclear membrane, LEM-domain proteins have been shown to play critical roles in various cellular processes such as DNA replication and regulation of gene expression. LAP2β regulates DNA replication by interacting with HA95 during the G1 phase of the cell cycle [12]. This interaction with HA95 leads the prereplication complexes to the replication origin and stabilizes it. Disruption of this interaction causes release of the prereplication complex components and triggers the proteolysis of Cdc6.

Pathological consequences have been described for LEM-domain proteins in genetic disorders in humans and are collectively called laminopathies [5], [13]. For example, Emerin deficiency causes Emery-Dreifuss Muscular Dystrophy (EDMD) [9], [14], [15] and MAN1 deficiency leads to osteopoikilosis, Buschke-Ollendorf syndrome and melorheostosis [16]. In addition to these laminopathies, involvement of LAP2 in carcinogenesis has been described. For example, LAP2β has been shown to be involved in proliferation of malignant lymphocytes [12], [17], [18]. Moreover, overexpression of LAP2α was reported in larynx, lung, stomach, breast and colon cancer tissues [19].

The LAP2 family of LEM domain proteins, is composed of at least six isoforms in mammals: α, β, γ, δ, ε, ζ, [6], [20], [21], [22]. These isoforms are generated by alternative splicing of the same transcript. All isoforms except the mammalian LAP2α and LAP2ζ are inner nuclear membrane proteins and share a similar domain organization. The N-terminal segment contains the LEM-domain and LEM-like domain. Unlike the LEM-domain, LEM-like domain can interact directly with chromatin without help of BAF. The C-terminal segment of LAP2 proteins has lamin-binding domains. Notably the C-terminal segment of α-isoform lacks a putative transmembrane domain, so the protein is distributed throughout the nucleus. Although LAP2α, β, and γ are expressed ubiquitously in the majority of mammalian cells, differential expression of LAP2 isoforms has been described. Differentiated tissues highly express the LAP2γ isoform, however, tissues with proliferating cells express more of the LAP2α and LAP2β isoforms [23].

Although its critical roles in genetic disorders and hematopoietic malignancies have been described, expression and roles of LAP2 in other cells or diseases are poorly characterized. In the present study, we found for the first time a novel role of LAP2β in regulation of motility of cancer cells and overexpression of LAP2 in diverse digestive tract cancers.

**Figure 1:**
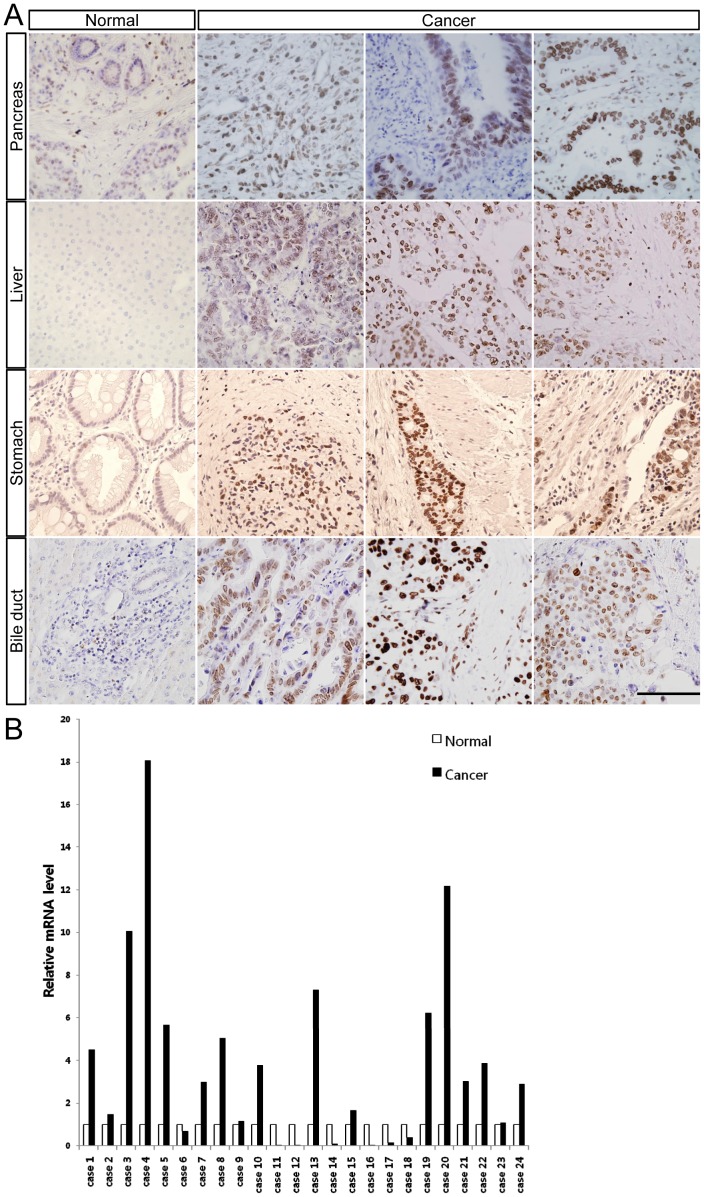
LAP2 is overexpressed in diverse digestive tract cancers. (A) Immunohistochemical staining showed overexpression of LAP2 in diverse digestive tract cancers including pancreas, liver, stomach and bile duct cancers. Note overexpression of LAP2 in metastatic cancer cells. Scale bar, 200 µm. (B) Overexpression of LAP2β in gastric cancer tissues was examined by real-time PCR using specific primers for the β-isoform. GAPDH was used to normalize all data.

**Figure 2:**
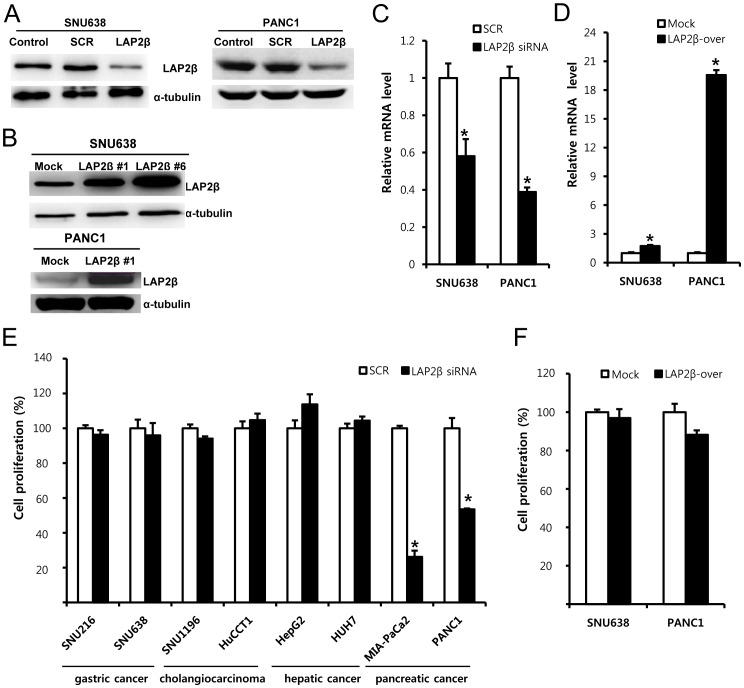
Role of LAP2β in the proliferation of cancer cells. Western blotting (A, B) and real-time PCR (C, D) were used to determine the efficiency of knockdown (A, C) or overexpression (B, D) of LAP2β in SNU638 or PANC1 cells. Data are the means±SD of three independent experiments (*P<0.01, Student’s t-test). (E) Effect of LAP2β knockdown on proliferation of cancer cells. WST-1 assay was used to measure proliferation of cancer cells in the presence of 10% FBS. Five days after transfection with by 100 nM LAP2β siRNA or 100 nM scrambled (SCR) siRNA, WST-1 proliferation assay was performed. (F) Effect of LAP2β overexpression on proliferation of cancer cells. WST-1 assay was conducted in SNU638 or PANC1 cells overexpressing LAP2β gene or control vector. Data are the means±SD of three independent experiments in quintuplicate (*P<0.01, Student’s t-test).

## Methods

### Cell Cultures and Transfection

Following digestive tract cancer cells were used; gastric cancer cells (SNU216, SNU638), cholangiocarcinoma cells (SNU1079, HuCCT1), pancreatic cancer cells (PANC1, MIA-PACA2), hepatocarcinoma cells (Huh7, HepG2). HuCCT1, HepG2, SNU216, SNU638, SNU1079 and Huh7 cells were cultured in RPMI1640 supplemented with 10% fetal bovine serum (FBS) and 100 µg/ml of penicillin/streptomycin. PANC1 and MIA-PACA2 cells were cultured in Dulbecco's Modified Eagle's Medium (DME)/high glucose supplemented with 10% FBS and 100 µg/ml of penicillin/streptomycin. Most cells were cultured at 37°C, 5% CO2 incubator. Cells were transfected with siRNA using DhamaFECT Reagent 3 (Dhamacon, Lafayette, CO, USA) according to the manufacturer’s instructions. The sequences of siRNA are as follows: LAP2β siRNA (Bioneer, Daejeon, Korea), 5′- ACA AGA GGG UCA AGA AGA A(dTdT) −3′ and 5′- UUC UUC UUG ACC CUC UUG U(dTdT) −3′; scrambled (SCR) siRNA (Dhamacon, Lafayette, CO, USA), 5′- GAU CCG CAA AAG AGC GAA A(dTdT) −3′ and 5′- UUU CGC UCU UUU CGC GAU C(dTdT) −3′.

### Overexpression of LAP2β

The DNA expression construct pCMV-SPORT6-LAP2β (Thermo scientific open biosystem, Huntsville, AL, USA) was used to drive overexpression and G418 (Sigma-Aldrich, St. Louis, MO, USA) was used for selection. Cells were co-transfected with pCMV-SPORT6-LAP2β/pIRES-Neo at a 5∶2 ratio using FuGENE HD (Roche, Nutley, NJ), in accordance with the manufacturer's instructions. MOCK cells were established simultaneously using empty control vector.

### Western Blotting

After gel electrophoresis and transfer to a PVDF membrane, the membrane was blocked for one hr. After adding primary antibodies (mouse anti-human LAP2β (BD Biosciences, San Jose, CA, USA, 1∶1000) and mouse anti-α-tubulin (BioGenex, 1∶10000)) in blocking solution the membrane was incubated in the primary antibody at 4°C overnight on a shaker. The appropriate secondary antibody was applied (1∶2000, horseradish peroxidase-conjugated anti-mouse) and incubated at room temperature for 2 h on a shaker. Lastly, the proteins were detected using LAS3000.

**Figure 3:**
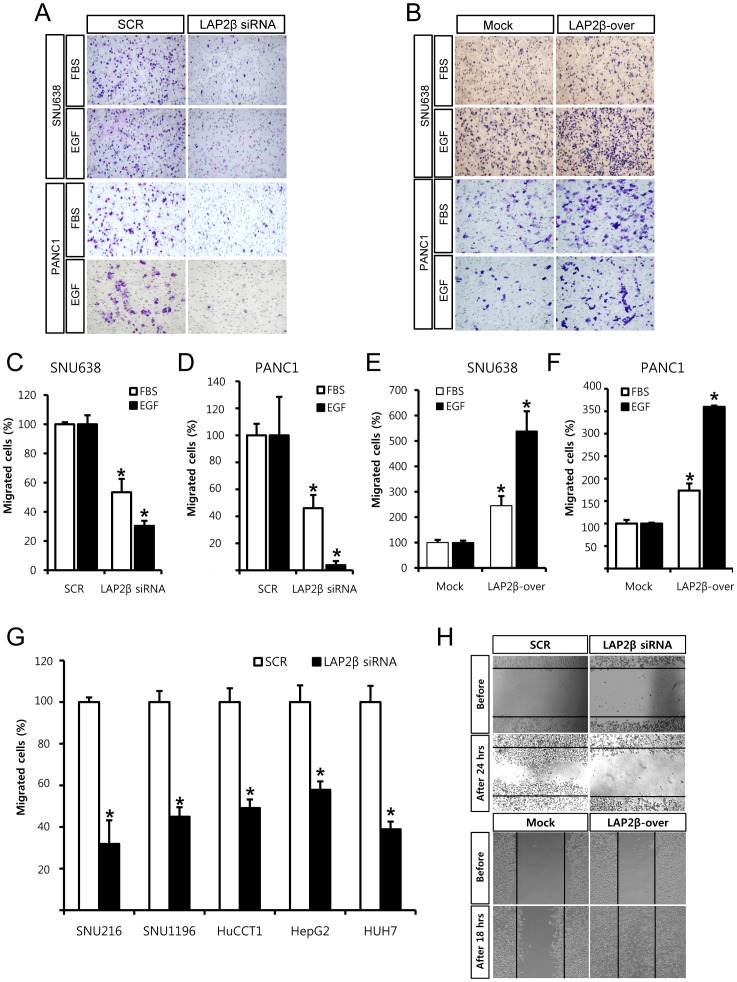
LAP2β regulates migration of diverse digestive tract cancers cells. Boyden chamber assay (A-G) and wound healing assay (H, SNU638 cells) were used to measure migration of cancer cells. LAP2β siRNA significantly inhibited FBS- or EGF-induced migration compared to SCR siRNA in SNU638 (A, C), PANC1 (A, D) or other digestive tract cancer cells (G). Overexpression of LAP2β in SNU638 (B, E) or PANC1 (B, F) cells significantly increased migration compared to the control vector (B, E, F). EGF (100 ng/ml) or 10% FBS was used to induce chemotaxis. Mitomycin C (0.01 µg/ml) was added to remove effects of proliferation. Two days after transfection with 100 nM LAP2β siRNA or 100 nM scrambled (SCR) siRNA, both migration assays were performed. Four or six hours later after addition of EGF or FBS into Boyden chamber assay, cells were fixed. After a scratch in wound healing assay, migrated cells were fixed at the indicated times. Representative staining of migrated cells was presented (A, B). Migrated cells were counted and the data are presented as graphs (C-G). Data are the means±SD of three independent experiments in triplicate (C-G, *P<0.01, Student’s t-test).

**Figure 4:**
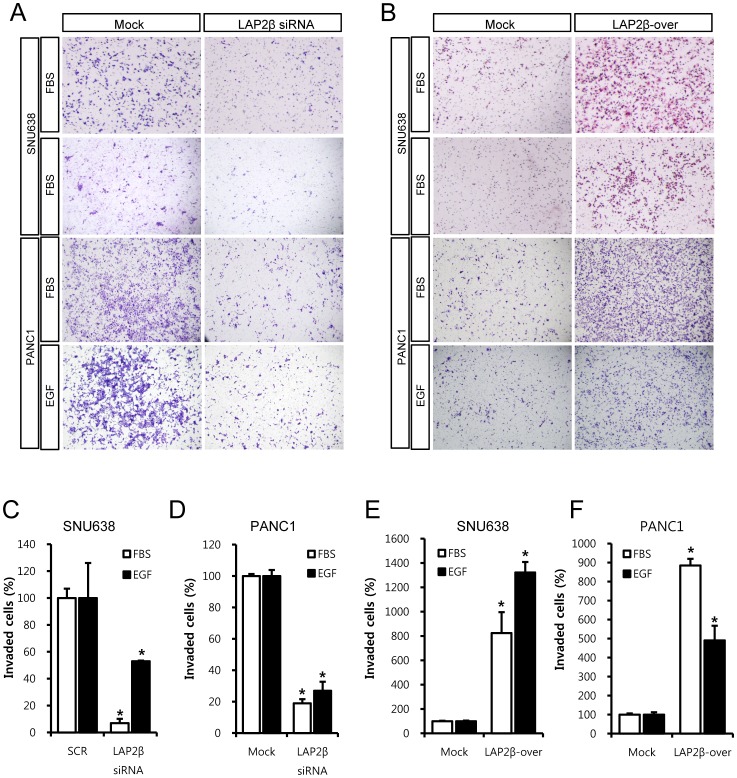
LAP2β regulates invasion of gastric and pancreatic cancer cells. Matrigel invasion assay was used to measure invasion of cancer cells. Knockdown of LAP2β significantly inhibited FBS- and EGF-induced invasion compared to SCR siRNA in SNU638 (A, C) or PANC1 (A, D) cells. Overexpression of LAP2β in SNU638 (B, E) or PANC1 (B, F) cells significantly increased invasion compared to the control vector. EGF (100 ng/ml) or 10% FBS was used to induce invasion. Mitomycin C (0.01 µg/ml) was added to remove effects of proliferation. Two days after transfection with 100 nM LAP2β siRNA or 100 nM scrambled (SCR) siRNA, the invasion assays were performed. Representative staining of invaded cells was presented (A, B). Invaded cells were counted and the data are presented as graphs (C-F). Data are the means±SD of three independent experiments in triplicate (C-F, *P<0.01, Student’s t-test).

### Real-time PCR

Gastric cancer tissues were obtained with written informed consent from patients who underwent surgical resection at Pusan National University Hospital and Pusan National University Yangsan Hospital, and the study was approved by the institutional review board of the hospitals (Permit Number 2009-13). Total RNA from tissues or cells were extracted using Trizol reagent (Invitrogen, Calsbad, CA, USA) or RNeasy Mini kit (Qiagen, Valencia, CA, USA) in accordance with the manufacturer’s protocol. cDNA was synthesized with MMLV reverse transcriptase (Promega, Madison, WI, USA), dNTP and oligo-dT primers. The primer sequences were as follows: LAP2β, 5′- AGG GCA GAG CAA AGA CTC CAG TAA CAA −3′ and 5′- TTA TTC CAG CTT GAG AAT AGC TCT GAT TGT GC −3′; MARCKS, 5′- GTG CCC AGT TCT CCA AGA CCG CA −3′ and 5′- GGC CAT TCT CCT GTC CGT TCG CT −3′, IL6; 5′- TAG CCG CCC CAC ACA GAC AGC C −3′ and 5′- TTC TGC CAG TGC CTC TTT GCT GCT −3′; STAT3, 5′- AAC ATG GCT GGC AAG GGC TTC TCC T −3′ and 5′- AGT GCT CAA GAT GGC CCG CTC CC −3′; GAPDH, 5′- GCA GCC TCC CGC TTC GCT CT −3′ and 5′- TGG TGA CCA GGC GCC CAA TAC G - 3′. Real-time RT-PCR was carried out using Power SYBR Green PCR Master Mix (Applied Biosystems, Foster city, CA, USA) in an ABI Prism 7500 sequence detector (Applied Biosystems, Foster city, CA, USA) in accordance with the manufacturer’s protocol. GAPDH was used to standardize cDNA input levels.

### Immunohistochemistry

After deparaffination and rehydration, the slides were subjected to 0.3% hydrogen peroxide for 30 min to quench endogenous peroxidase activity. Blocking was done with 10% normal donkey serum (NDS) and 1% BSA in 1XPBS. Then the slides were incubated overnight at 4°C in blocking buffer containing following primary antibody; anti-human LAP2 (1∶200, BD Biosciences, San Jose, CA, USA), anti-STAT3 (1∶100, Cell Signaling, Beverly, MA,USA), anti-IL6 (1∶100, ABCAM, Cambridge, UK) or anti-MARCKS (1∶50, Santa Cruz Biotechnology, Santa Cruz, CA, USA) antibody. Secondary antibody (horseradish peroxidase-conjugated) binding was done at a 1∶200 dilution in blocking buffer for 2 h at RT. Detection was performed with HRP (Vector Laboratories) by using the DAB substrate kit (Vector Laboratories). Counterstaining was done for 1 min with hematoxylin staining buffer (Sigma–Aldrich, St. Louis, MO).

### Cell Proliferation Assay

Two, three or five days following transfection with siRNA, we added 10 µl of pre-mixed water-soluble tetrazolium salt-1 (WST-1, Roche, Indianapolis, IN, USA) cell proliferation reagent into each well. These cells were incubated for two hr in the incubator. Cell viability was measured by absorbance at 450 nm using an ELISA reader (TECAN, Mannedorf, Switzerland).

### Boyden Chamber Assay

Migration of cancer cells was measured in a Boyden chamber. Approximately 5×10^4^ cells in 0.05 ml of serum-free RPMI1640 medium were seeded to the well membrane–coated with Type I collagen. To remove effects of proliferation, mitomycin C (0.01 µg/ml, Sigma, USA) was added. Cells were allowed to migrate for four or six hrs. Membranes were fixed and stained using Diff-quik solution (Sysmex, Kobe, Japan) for one min and washed with distilled water. Cell number in 10 randomly chosen fields was determined using a light microscope. Experiments were performed in triplicate and repeated thrice.

### Wound Healing Assay

The cell monolayer was scratched with a yellow pipet tip and migration of cells to the wounded area was observed under an inverted microscope. To remove effects of proliferation, mitomycin C (0.01 µg/ml, Sigma, USA) was added. Images were taken at the indicated times. Measurements were taken from five individual microscopic fields in each experiment, and the representative data from three experiments are presented.

### Matrigel Invasion Assay

The ability of cancer cells to invade was determined using 24-well BioCoatTM MatrigelTM chamber inserts (BD Biosciences, San Jose, CA, USA). The inner insert of the invasion chambers were coated with 0.5 mg/ml growth factor-reduced Matrigel (BD Biosciences, San Jose, CA, USA) and the outer insert by 0.5 mg/ml of fibronectin (Sigma-Aldrich, St. Louis, MO, USA). Cells were seeded into inserts at a density of 5×10^4^ per insert in serum-free medium and then transferred to wells filled with the culture medium containing 10% FBS or 100 ng/ml EGF as a chemoattractant. To remove effects of proliferation, mitomycin C (0.01 µg/ml, Sigma, USA) was added. After 24 (10% FBS) or 52 hrs (100 ng/ml EGF) of incubation, non-invading cells on the top of the membrane were removed by scraping. Invaded cells on the bottom of the membrane were fixed, followed by staining with Diff-quik solution. Experiments were performed in triplicate, and at least 10 fields were counted in each experiment.

### Liver Metastasis Xenograft Model

The ability of the transfectants to metastasize to the liver was evaluated by slowly injecting cells (5×10^6^/0.05 ml) into the spleen of nude mice via a 27-gauge needle (NK4 group, mock group; n = 5/group). For pathological studies, all mice were killed at 5 weeks and livers were isolated, fixed in 10% neutral - buffered formalin, and embedded in paraffin. Sections were stained with hematoxylin and eosin. This study was carried out in strict accordance with the recommendations in the Guide for the Care and Use of Laboratory Animals of the National Institutes of Health. The Pusan National University Institutional Animal Care and Use Committee (PNUIACUC) approved the experimental procedures (Permit Number: PNU-2010-00083).

### cDNA Microarray

Total RNA was extracted from SNU638-LAP2 and SNU638 mock cells using RNeasy Mini kit (Qiagen, Valencia, CA, USA) in accordance with the manufacturer’s protocol. Quantified RNA was then used for microarray analysis on Human HT-12_v4_Bead chips (Illumina Inc., San Diego, CA, USA). Total RNA samples were labeled using the Illumina TotalPrep RNA Amplification Kit (Ambion, Applied Biosystem, CA, USA) for cDNA synthesis and in vitro transcription. Single-stranded RNA (cRNA) was generated and labeled by incorporating biotin-NTP (Ambion). A total of 0.5 µg of biotin-labeled cRNA were hybridized at 58°C for 16 h to Illumina’s Human HT-12_v4_BeadChip (Illumina). The hybridized biotinylated cRNA was detected with streptavidin-Cy3 and quantified using a BeadArray Reader Scanner (Illumina) according to the manufacturer’s instructions. Array data were processed and analyzed by Illumina BeadStudio version 3.0 software (Illumina). Scanned data were normalized by the quantile-quantile normalization method and log-transformed (by base two). All data are MIAME compliant and the raw data were submitted to the public repository (NCBI’s GEO Accession Number: GSE31450).

**Figure 5:**
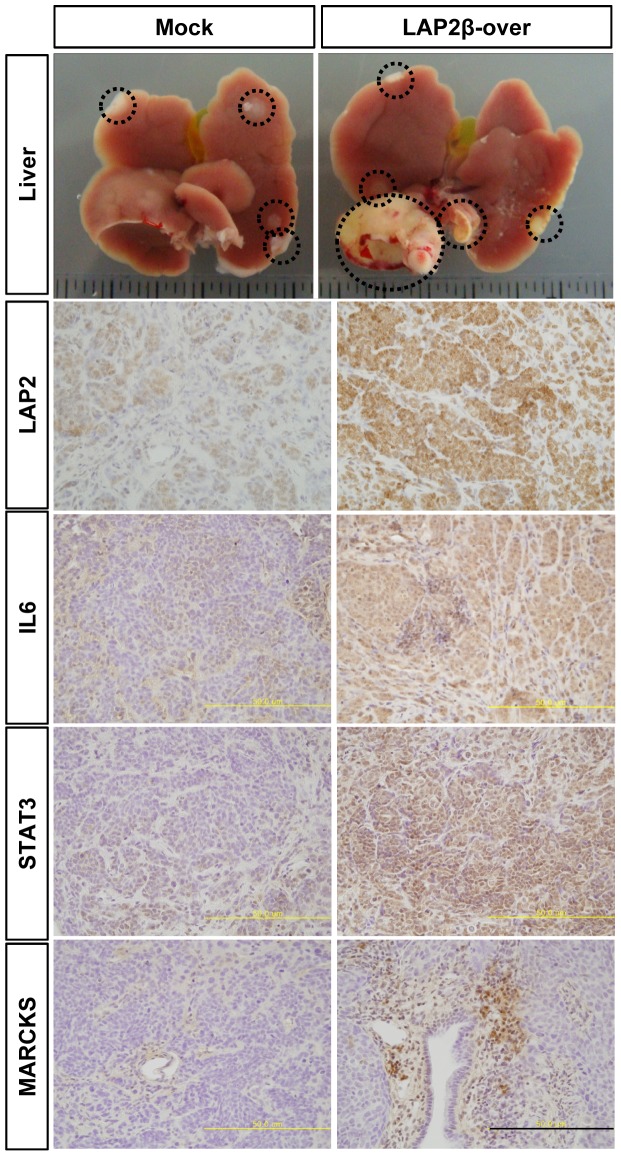
LAP2β enhances metastatic efficiency in a xenograft model. Gastric cancer cells overexpressing LAP2β gene or control vector were injected into spleen and metastasis to liver was examined 5 weeks later. Metastatic tumor regions were indicated by dotted circles. Representative immunostainings with anti-LAP2, anti-IL6, anti-STAT3, and anti-MARCKS antibodies, and H&E stainings in a liver metastasis are presented. Asterisk indicates tumor lesions. Scale bar, 50 µm.

### Data Analysis

All data are presented as means±SD. The difference between the mean values of two groups was evaluated using the Student’s t-test (unpaired). For comparison of more than 3 groups, a one-way analysis of variance (ANOVA), followed by Tukey’s multiple comparisons were used. *Indicates a P value of <0.05, which was considered statistically significant.

## Results

### LAP2 is Widely Overexpressed in Diverse Digestive Tract Cancers

To examine expression patterns of LAP2 in digestive track cancers including stomach, pancreas, liver, and bile duct cancer, we carried out immunohistochemistry using patient tissues (n = 15 per each cancer type). LAP2 protein was widely overexpressed in the cancerous area of tissues compared to non-cancerous areas (Fig. 1A, 47% in stomach cancer, 27% in pancreas cancer, 30% in liver cancer, 40% in bile duct cancer). Notably, expression of LAP2 was observed in metastatic cancer cells of patients’ tissues. Because LAP2 has numerous isoforms, we focused on LAP2β. To confirm the results of immunohistochemistsry, we performed real-time PCR using LAP2β-specific primers in gastric cancer tissues. Although all tested tissues did not overexpress LAP2β, it was overexpressed in 13 cases (total 24 cases, Fig. 1B).

**Table 1:** LAP2β-induced change in gene expression.

UP			DOWN		
Probe ID	Target ID	FC	Probe ID	Target ID	FC
4830541	HBE1	13.43	1780482	CACHD1	0.78
430446	KRT81	10.02	4810128	PHLDA2	0.67
2630240	SPINK6	9.22	870338	EGR1	0.65
6590132	IGFBP3	5.71	2570300	IFI44	0.51
2600133	ALPP	4.11	4640086	FOXQ1	0.50
1980132	SPINK6	4.04	4900561	PSG1	0.49
5570768	HOXA11AS	3.15	2060040	ADORA2B	0.48
7210192	ADA	2.90	1580435	TGM2	0.46
610437	CD24	2.49	2970154	KLK6	0.45
2940746	NNMT	2.24	6940475	AKAP12	0.44
730739	TCEAL3	2.05	2370450	ASPH	0.44
3140619	OR51B5	2.03	5860039	CALB2	0.44
6060484	MARCKS	1.94	670041	AKAP12	0.44
510097	HOXA10	1.85	1580246	LOC399959	0.40
4150014	CEACAM1	1.81	2060477	AGPAT9	0.34
4040576	IL6	1.69	6330270	GPC4	0.30
5090619	STAT3	1.50	3190112	SERPINB1	0.26

FC, fold change.

### Roles of LAP2β in Proliferation, Migration, and Invasion of Cancer Cells

To examine roles of LAP2β in carcinogenesis, we knocked-down or overexpressed LAP2β using siRNA or cDNA, respectively. We checked the efficiency of the modulation of LAP2β gene by western blotting or real-time PCR (Fig. 2A-D). LAP2β siRNA (100 nM) decreased the mRNA level of LAP2β in SNU638 or PANC1 cells compared to SCR siRNA by 42% or 61%. Overexpression of LAP2β by cDNA transfection increased the mRNA level of LAP2β in SNU638 or PANC1 cells (clone#6) compared to the control vector by 1.7 or 19.6 fold respectively.

Next, we examined the role of LAP2β in proliferation of cancer cells. Five days after transfection with SCR or LAP2β siRNA, WST-1 proliferation assay was carried out. Knockdown of LAP2β did not affect proliferation of most tested cancer cells except pancreatic cancer cells (Fig. 2E). LAP2β siRNA inhibited proliferation of MIA-PaCa2 and PANC1 pancreatic cancer cells compared to SCR siRNA by 74% and 46% respectively (Fig. 2E). We observed similar results when we performed WST-1 proliferation assay two or three days after the transfection. Overexpression of LAP2β in SNU638 or PANC1 cells slightly affected proliferation (Fig. 2F).

To determine the role of LAP2β in migration of cancer cells, we conducted studies using a Boyden chamber assay. In all tested cancer cells, knockdown of LAP2β inhibited migration of cancer cells (Fig. 3). For example, LAP2β siRNA inhibited FBS- or EGF-induced migration of SNU638 cells compared to SCR siRNA by 47% and 70% respectively (Fig. 3A, & 3C). In constrast, overexpression of LAP2β increased FBS- and EGF-induced migration of SNU638 cells compared to mock cells by 145% and 387% respectively (Fig. 3B & 3E). Similar results were obtained in LAP2β- overexpressing PANC1 cells (Fig. 3B & 3F). This effect on migration of cancer cells was further confirmed by a wound healing assay in SNU638 cells (Fig. 3H). These results led us to examine the role of LAP2β in the invasion of cancer cells. In a Matrigel invasion assay, LAP2β siRNA inhibited FBS- and EGF-induced invasion of SNU638 cells compared to SCR siRNA by 93% and 47% respectively (Fig. 4A & 4C). Similar results were obtained in PANC1 (Fig. 4A & 4D) or SNU216 (data not shown) cells. In contrast, overexpression of LAP2β increased FBS- and EGF-induced invasion of SNU638 cells compared to control vector by 725% and 1,223% respectively (Fig. 4B & 4E). Similar results were obtained in PANC1 (Fig. 4B & 4F) cells.

### LAP2β Enhances Metastatic Efficacy of Gastric Cancer Cells in a Liver Metastasis Xenograft Model

Regulation of the motility of cancer cells by LAP2β suggested the possibility that LAP2β regulates metastasis of cancer cells in vivo. To examine this possibility, we injected gastric cancer cells into spleen of nude mice and then observed metastasis in the liver. Interestingly, overexpression of LAP2β increased the efficiency and the size of liver metastasis (Fig. 5) and mortality of tested mice. 67% of mice injected with gastric cancer cells overexpressing LAP2β died 8 weeks later after the injection, while all control mice injected with gastric cancer cells expressing control vector survived. In the histological examination of xenograft tissues, we confirmed overexpression of LAP2β in the xenograft derived from mice injected with LAP2β-overexpressing cells (Fig. 5).

### LAP2β-induced Changes in mRNA Expression

**Figure 6:**
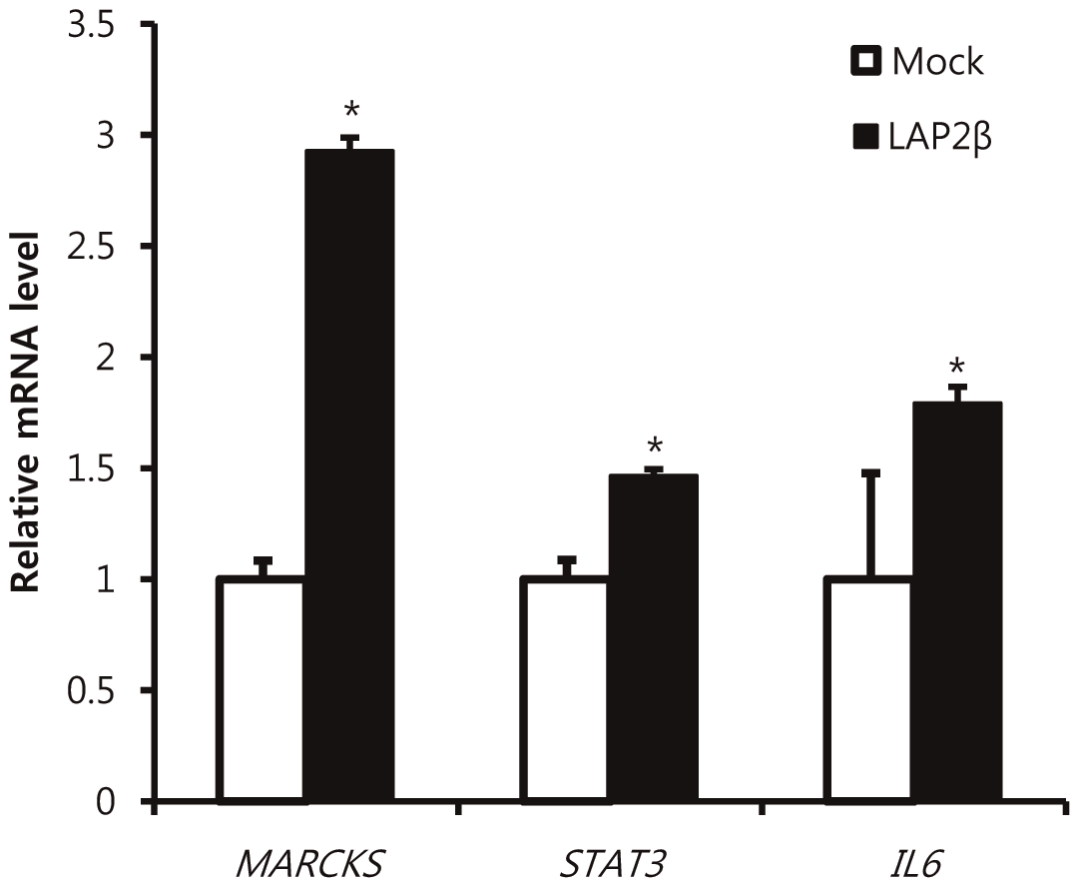
LAP2β-induced gene expression. Gene expression between gastric cancer cells overepxressing LAP2β gene or control vectors were compared by cDNA microarray. Real-time PCR was used to confirm the LAP2β-induced change in gene expression of *MARCKS, STAT3 and IL-6* in SNU638 cells. The data are plotted as fold changes compared with mock cells. Data are the means±SD of three independent experiments in quintuplicate (*P<0.01, Student’s t-test).

To reveal the underlying mechanism of LAP2β-regulated motility, we performed a cDNA microarray (NCBI’s GEO Accession Number: GSE31450). Although the mRNA level of LAP2β was overexpressed in the stable cell line about 1.7 fold, those of many genes were changed by the overexpression (Fig. 2 & Table 1). Among the significantly changed genes by LAP2β, we focused on myristoylated alanine-rich C kinase substrate (MARCKS), signal transducer and activator of transcription3 (STAT3) and interleukin6 (IL6) because these genes have been reported to regulate motility of cells. Real-time PCR for each gene confirmed significant changes in mRNA levels of each gene (Fig. 6). Overexpression of LAP2β increased the mRNA levels of MARCKS and IL6 compared to control vector by 193% and 79% respectively (Fig. 6). Moreover, increased expressions of MARCKS, IL6 and STAT3 were observed in the xenograft derived from mice injected with LAP2β-overexpressing cells (Fig. 5).

## Discussion

LAP2, one of LEM domain proteins, has been mainly described to play a structural role in the nuclear membrane and to be involved in several genetic disorders. However, here we present for the first time its expression and roles in diverse digestive tract cancers. In particular, we found that LAP2β can control motility of cancer cells as well as contribute to metastasis of cancer cells.

Metastasis of cancer cells greatly affects the prognosis of cancer patients. Several results in the present study support that LAP2β regulates the motility and metastasis of cancer cells. In vitro experiments in the Boyden chamber, wound healing and Matrigel invasion assays, showed that knockdown decreased while overexpression of LAP2β increased the migration and invasion of cancer cells (Fig. 3 & 4). Moreover, in the xenograft model, LAP2β enhanced metastasis of cancer cells (Fig. 5). Although control vector-transfected cells caused metastasis in the xenograft model, the effect was quite inefficient and slow. In contrast, LAP2β-overexpressed cells showed a more aggressive behavior in the xenograft. Furthermore, we found overexpression of LAP2 in metastatic cancer cells of tissues from patients (Fig. 1).

How can LAP2β contribute to motility and metastasis of cancer cells? We found several genes which were induced by LAP2β in the cDNA microarray analysis (Table 1), which was further confirmed by real-time PCR (Fig. 6) and immunohistochemistry in xenograft (Fig. 5). One of them, MARCKS, is responsible for the binding and cross-linking of actin filaments directly to the membrane [24]. Overexpression of MARCKS has been found in various cancers including hepatocellular carcinoma [25], pancreatic cancer [26], glioblastoma [27] and cholangiocarcinoma [28]. Moreover, MARCKS plays a critical role in EGFR-induced invasion of glioblastoma cells [29]. Many other studies have been shown the involvement of MARCKS in cellular motility [30].

Another candidate gene which mediates LAP2β-induced motility is IL-6, which is primarily produced during acute and chronic inflammation. Cancer cells that are exposed to IL-6 or secrete the cytokine as an autocrine factor show increased invasiveness [31], [32], [33]. Moreover, the inactivation of gp130, a transducer of IL-6 signaling, reduced the aggressiveness of breast cancer cells in vivo [34]. Several IL-6 signaling pathway-related genes including STAT3 are also associated with migration and invasion of cancer cells [35], [36], [37]. IL-6 is widely expressed in many solid cancers including prostate [38], breast [39], lung [40] cancer, and glioblastoma [41].

How can LAP2β regulate gene expression? LEM-domain proteins have been shown to be able to regulate gene expression by sequestering transcriptional regulators to the nuclear lamina. MAN1 binds to receptor-regulated R-Smads and antagonizes signaling by transforming growth factor β (TGFβ), activin and bone morphogenic protein (BMP) [42]. MAN1 deficiency leads to embryonic vascular remodeling defects in mice and bone development in humans [16], [43]. Another example is emerin binding to β–catenin, a downstream target of Wnt signaling, which promotes its exit from the nucleus [44]. Emerin deficiency leads to nuclear accumulation of β-catenin. LAP2β has been shown to interact with HDAC3 and regulate activity of E2F, p53 and NF-κB transcription factors [5], [45]. In future studies, how LAP2β can regulate MARCKS or IL-6 expression warrants further investigation.

The involvement of LAP2β in replication was suggested by a study in which truncated LAP2β altered DNA replication efficiency [18]. The regulation of DNA replication by LAP2β has been suggested to be mediated by two possible pathways. LAP2β can reduce the activity of E2F complex alone or with germ cell less (GCL) [46]. The other pathway is through interaction with HA95 during the G1 phase of the cell cycle [12]. This interaction with HA95 contributes to the stability of the prereplication complexes. In the present study, knockdown of LAP2β did not affect proliferation of most digestive tract cancer cells except pancreatic cancer cells (Fig. 2). Moreover, overexpression of LAP2β did not cause significant change in proliferation (Fig. 2), suggesting the regulation of proliferation by LAP2β in digestive tract cancer cells is not so critical.

Widespread overexpression of LAP2 in various digestive tract cancers is described for the first time in the present study (Fig. 1). Expression of LAP2β has been described in various normal tissues including skin, thymus, lung, testis and ovary. However, its expression in normal gastrointestinal tract was rarely detected [47]. Overexpression of LAP2β was reported in various hematological malignant cells and neuroblastoma cells [17], [48]. Moreover, LAP2α is overexpressed in various solid cancers including larynx, lung, stomach, breast and colon cancer [19]. Interestingly, the LAP2 promoter is reported to be regulated by the transcription factor, E2F [19].

In the present study, we found that LAP2 is widely overexpressed in digestive tract cancer cells and plays critical roles in motility of cancer cells. Although the detailed underlying mechanism for regulation of motility needs to be examined in future studies, these data suggest that LAP2β may be a possible target for therapeutics and diagnostics.
